# Adrenocortical Tumor Associated With Pathogenic Variant in KCNJ5 and DNA Methylation of CYP11B2 in Primary Aldosteronism

**DOI:** 10.1210/jcemcr/luae119

**Published:** 2024-07-18

**Authors:** Ko Aiga, Mitsuhiro Kometani, Masashi Demura, Takashi Yoneda

**Affiliations:** Department of Health Promotion and Medicine of Future, Kanazawa University Graduate School of Medicine, Kanazawa, Ishikawa 920-8641, Japan; Department of Health Promotion and Medicine of Future, Kanazawa University Graduate School of Medicine, Kanazawa, Ishikawa 920-8641, Japan; Department of Hygiene, Kanazawa University School of Medicine, Kanazawa, Ishikawa 920-8640, Japan; Department of Health Promotion and Medicine of Future, Kanazawa University Graduate School of Medicine, Kanazawa, Ishikawa 920-8641, Japan

**Keywords:** primary aldosteronism, KCNJ5, CYP11B2, DNA methylation

## Abstract

Primary aldosteronism (PA) is a subtype of secondary hypertension categorized as either unilateral PA (eg, aldosterone-producing adenoma [APA]) or bilateral PA. CYP11B2, an aldosterone synthase, is highly expressed in APA. Recent studies have revealed a high prevalence of pathogenic variants in *KCNJ5* and the role of DNA methylation on CYP11B2 in APA. We present a case of unilateral PA with pathogenic variants in *KCNJ5* and suppressed CYP11B2 expression. A 55-year-old woman with hypertension was referred to our hospital. A high aldosterone-renin ratio was observed; PA was confirmed using the captopril challenge test and the furosemide upright test. Although computed tomography showed no evident tumors in either adrenal gland, adrenal vein sampling revealed left gland dominance. Postoperatively, the aldosterone-renin ratio decreased and captopril challenge test showed negative findings. Pathogenic variants in the *KCNJ5* were detected in the adenoma. Although immunohistochemistry for CYP11B2 was negative in adenoma, an aldosterone-producing cell cluster was confirmed in the adjacent left adrenal gland. Furthermore, DNA methylation analysis of the adenoma indicated hypermethylation in the CYP11B2 promoter region. The pathogenic variant in *KCNJ5*, specific to APA, induces CYP11B2 overexpression, resulting in excess aldosterone. However, these effects can be suppressed by DNA methylation.

## Introduction

Primary aldosteronism (PA) is a common form of secondary hypertension, which is characterized by hypersecretion of aldosterone. There is an increased risk of cardiovascular morbidity and mortality in PA because of excess aldosterone production (1). Based on etiology, PA is divided into 2 subtypes: unilateral PA, such as aldosterone-producing adenoma (APA), and bilateral PA, often referred to as idiopathic adrenal hyperaldosteronism (1). Additionally, adrenalectomy is the preferred treatment for APA because of its potential curative effect (1). Conversely, medications are administered lifelong for idiopathic adrenal hyperaldosteronism (1).

In the past decade, the relationship between APA, gene mutations, and DNA methylation has been explored (2, 3). Pathogenic variants in *KCNJ5* induce the upregulation of CYP11B2 expression, an aldosterone synthase, leading to increased aldosterone production (4). Moreover, hypomethylation of the CYP11B2 promoter promotes the expression of CYP11B2 (2).

In this report, we present a case of adrenocortical adenoma in which a pathogenic variant in *KCNJ5* was detected in the nodule of the resected tissue; however, it did not show positive CYP11B2 immunostaining. The DNA methylation status was further analyzed to explore the underlying molecular mechanisms.

## Case Presentation

A 55-year-old woman with hypertension was referred to our department. At age 40 years, her blood pressure was 120 to 130/80-90 mm Hg. Thirteen years later, her systolic blood pressure began to rise to approximately 150 to 160 mm Hg. However, at age 55 years, the patient was hospitalized for cochlear implant surgery. Her systolic blood pressure remained elevated at 140 mm Hg during hospitalization. She was then referred to the endocrinology department for further evaluation.

## Diagnostic Assessment

**Figure 1.**
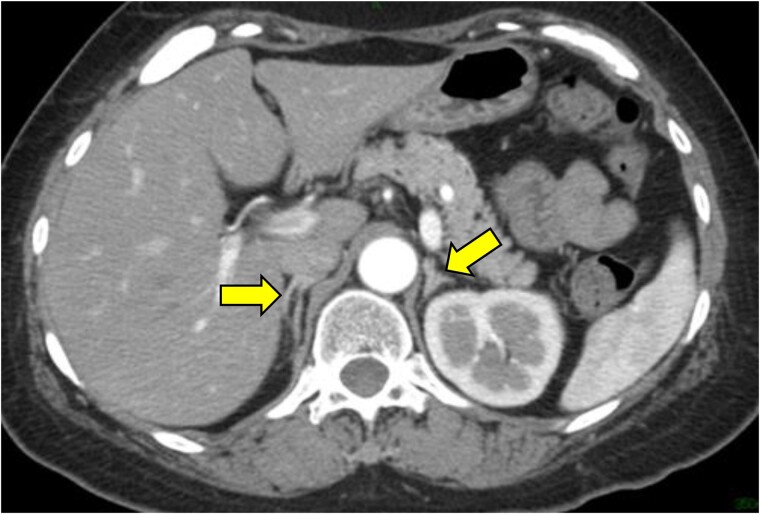
Abdominal computed tomography (CT) imaging. No apparent tumor was observed from the CT imaging. The yellow arrows indicate adrenal glands.

The patient was a social drinker with a smoking history of an average of 10 cigarettes per year when she was aged 20 to 23 years. She had a medical history of appendicitis at age 10 years. Her family history included hypertension and depression in her mother and liver cirrhosis in her father. She was on a prescription of calcium channel blockers (azelnidipine 16 mg) when she was referred to our hospital. On physical examination, the patient's height, weight, body mass index, blood pressure, pulse, and body temperature were 153 cm, 64 kg, 27 kg/m^2^, 149/92 mm Hg, 65 beats/min, and 36.8 °C, respectively. The laboratory data revealed plasma renin activity (PRA) suppression at 0.1 ng/mL/h (normal value: 0.2-2.7 ng/mL/h) and an elevated plasma aldosterone concentration (PAC) level at 19.1 ng/dL (normal value: 2-13 ng/dL). However, hypokalemia was not observed. The captopril challenge test (CCT) and furosemide upright test were performed, and both tests were positive (Table 1). Following the Japan Endocrine Society PA guidelines, the cutoff value of the CCT and furosemide upright test were assigned by the aldosterone-renin ratio: 200 (after 90 minutes) and PRA: 2.0 ng/mL/h (after 120 minutes), respectively (1). Based on these confirmatory tests, the patient was diagnosed with PA. Computed tomography (CT) was conducted to investigate the lateralization of the PA. However, no apparent lesions were observed in the CT images (Fig. 1). Adrenal vein sampling (AVS) without ACTH stimulation was performed to further clarify PA localization with the successful selection of both adrenal veins. Per the PA guidelines, the cutoff values of the selectivity index (SI), lateralization index (LI), and contralateral ratio (CR) were assigned as 2, 2, and 1, respectively (1, 5). SI, LI, and CR were used to evaluate the cannulation of the catheter, lateralization, and suppression of the nondominant glands, respectively. The results revealed that aldosterone was predominantly produced in the left adrenal gland (Table 2). However, the CR did not show the suppression of the nondominant adrenal gland.

**Table 1.** Confirmatory tests

Preoperative	Baseline	90-minutes after challenge	120 in after challenge
FUT	PRA <0.1 (ng/mL/h) PAC: 9.4 (ng/dL)		PRA 0.3 (ng/mL/hr) PAC 17.7 (ng/dL)
CCT	PRA: 0.5 (ng/mL/h) PAC: 10.3 (ng/dL)	PRA: 0.2 (ng/mL/h) PAC: 6.6 (ng/dL)	—
Post-operative	Baseline	90 minutes after challenge	120 minutes after challenge
CCT	PRA: 0.4 (ng/mL/h) PAC: 8.8 (ng/dL)	PRA: 0.4 (ng/mL/h) PAC: 7.7 (ng/dL)	

Abbreviations: ARR, aldosterone-to-renin ratio; CCT, captopril challenge test; FUT, furosemide upright test; PAC, plasma aldosterone concentration; PRA, plasma renin activity.

**Table 2.** Adrenal vein sampling without ACTH stimulation

Location	PAC	F	PAC/F	SI	LI	CR
IVC	7.3 (ng/dL)	8 (µg/dL) [221 (nmol/L)]	0.91 (ng/dL)/(µg/dL) [0.033 (ng/dL)/(nmol/L)]	—	—	3 (RAV/IVC)
RAV	294 (ng/dL)	107 (µg/dL) [2952 (nmol/L)]	2.75 (ng/dL)/(µg/dL) [0.10 (ng/dL)/(nmol/L)]	13	2.7 (LAV/RAV)	
LAV	424 (ng/dL)	57 (µg/dL) [1573 (nmol/L)]	7.44 (ng/dL)/(µg/dL) [0.27 (ng/dL)/(nmol/L)]	7		—

Abbreviations: CR, contralateral ratio; F, plasma cortisol concentration; IVC, inferior vena cava; LAV, left adrenal vein; LI, lateralization index; PAC, plasma aldosterone concentration; RAV, right adrenal vein; SI, selectivity index.

## Treatment

Based on the AVS results, the patient chose surgical treatment. Ninety-seven days before surgical treatment, the patient was prescribed 25 mg of eplerenone daily. A left adrenalectomy was performed.

## Outcome and Follow-up

**Figure 2.**
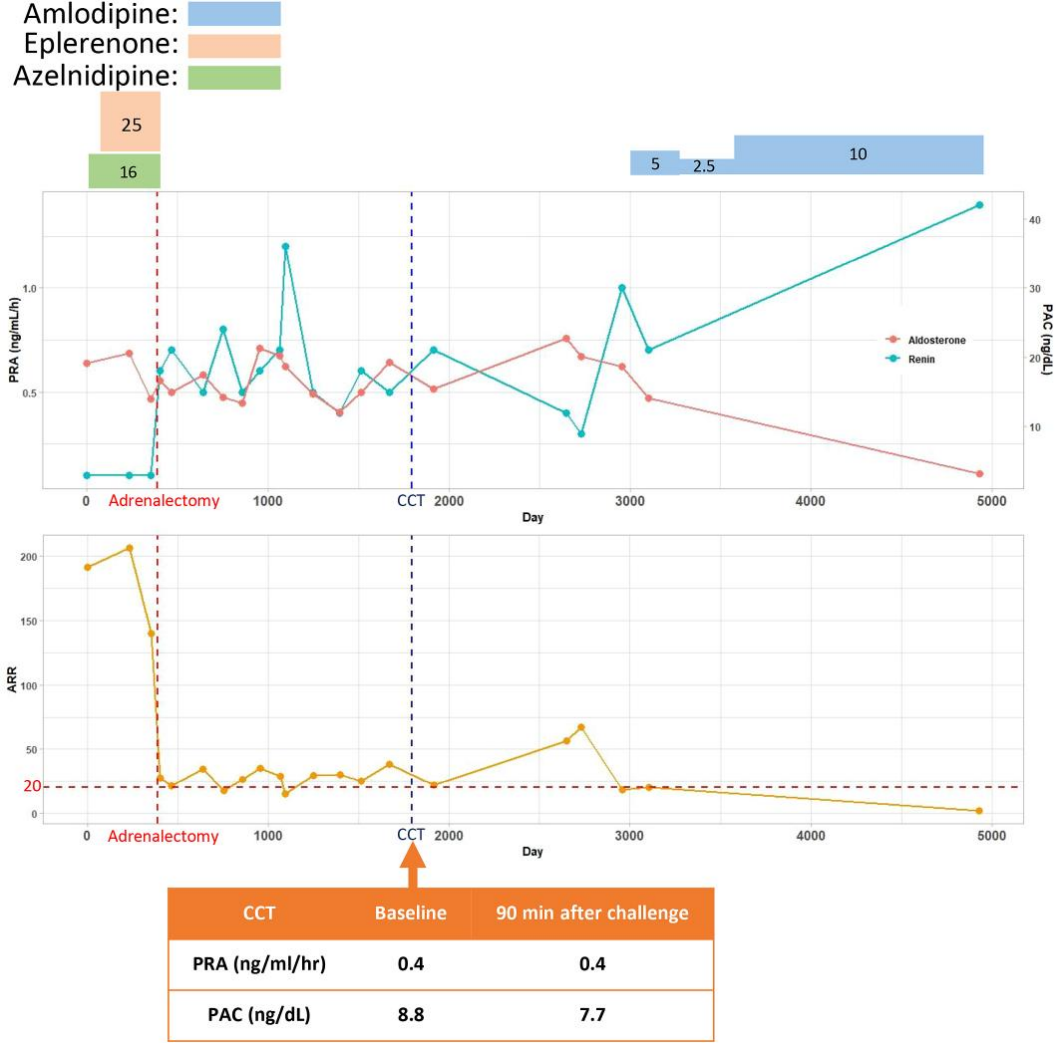
The timeline of adrenal hormones and captopril challenge test. Blue, orange, and green boxes represent amlodipine, eplerenone, and azelnidipine, respectively. The numbers inside the box show the quantity of medicine (units: mg/day for amlodipine, eplerenone, and azelnidipine, respectively). Red and blue dotted lines represent adrenalectomy and captopril challenge test results, respectively.

**Figure 3.**
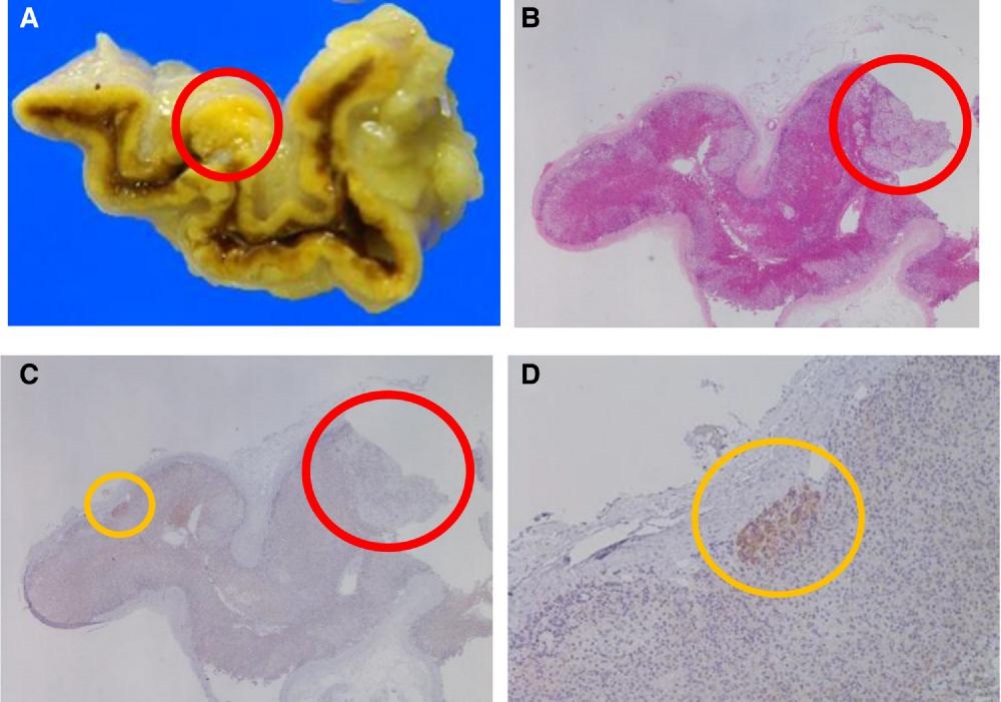
Pathological analysis. Gross pathological finding. The red circle indicates the 2-mm-sized nodule. (A) Microscopic finding (low magnification) with hematoxylin and eosin staining. The red circle indicates the nodule. (B) Microscopic finding (low magnification) with CYP11B2 immunohistochemistry results. (C) The red and orange circles indicate the nodule and aldosterone-producing cell cluster (APCC), respectively. Microscopic finding (high magnification) with CYP11B2 immunohistochemistry results. The orange circle indicates the APCC (D).

**Figure 4.**
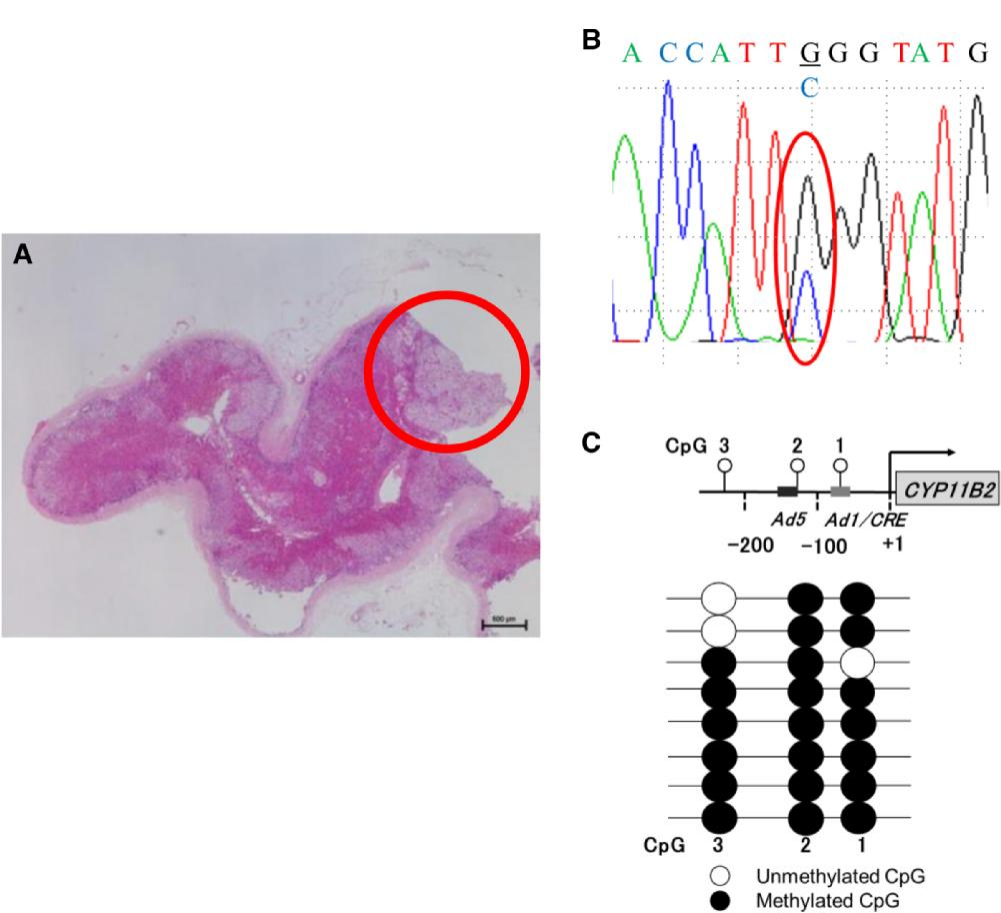
Gene and methylation analysis. Sanger sequencing and DNA methylation analysis were performed at the region of the red circle (nodule). (A) A pathogenic variant in *KCNJ5* was detected in the nodule. The amino acid substitution was G151R. (B) DNA methylation analysis using a DNA methylation activity assay. White/black circles represent unmethylated and methylated CpG sites, respectively. The promoter region of CYP11B2 in the nodule was also investigated. (C).

After adrenalectomy, PRA, PAC, and aldosterone-renin ratio improved, and CCT was negative 1400 days after the surgery (Fig. 2). Histopathological and genetic analyses were performed on the resected tissues. Macroscopic findings and hematoxylin and eosin staining revealed the presence of a 2-mm nodule in the resected tissue (Fig. 3). Genetic analysis of the nodule revealed a pathogenic variant in *KCNJ5* (G151R) (Fig. 4). Notably, CYP11B2 immunostaining was negative for the nodule (Fig. 3). Furthermore, CYP11B2 immunostaining revealed the presence of an aldosterone-producing cell cluster (APCC) in the vicinity of the nodule (Fig. 3). To explore the mechanisms underlying the low expression of CYP11B2 in the nodule, we investigated the DNA methylation status in the promoter region of CYP11B2. The detailed method for DNA methylation analysis has previously been described by Demura et al (6). The analysis was performed to evaluate the methylation rates of 3 CpG sites upstream of the promoter region of CYP11B2 (Fig. 4). The rates of methylation in CpG1, CpG2, and CpG3 were 75% (6/8), 100% (8/8), and 87.5% (7/8), respectively. Two cis-acting regulatory elements, Ad1 and Ad5, are located in CpG1 and CpG2, respectively.

## Discussion

APA is known to express elevated levels of CYP11B2, shown through positive immunohistochemistry for CYP11B2 (4). Pathogenic variants in *KCNJ5* exhibit the highest prevalence among APA-driving genes in Japan, accounting for 73% of all APAs (3). In the present case, we detected a pathogenic variant in *KCNJ5* in the adrenocortical adenoma. Therefore, this case was initially considered a typical APA. However, histopathological analysis was negative for CYP11B2 expression in the nodule, but an APCC was confirmed in the adjacent adrenal gland. Furthermore, methylation of the promoter region of CYP11B2 was confirmed. This study is the first to investigate the relationship between DNA methylation and *KCNJ5* mutant nodules with CYP11B2 negative immunostaining.

Recently, the criteria for confirmatory tests have been updated in Japan because of the replacement of the radioimmunoassay with the chemiluminescent enzyme immunoassay for PAC measurement. The present case was diagnosed and treated before the introduction of the chemiluminescent enzyme immunoassay method for PAC measurement. Further, the criteria for AVS without ACTH stimulation and confirmatory tests were followed as per the Japan Endocrine Society PA guidelines and the Endocrine Society PA guidelines, respectively. In the present case, the LI showed a slightly higher value than the cutoff value, which led to the left adrenalectomy. Conversely, the CR showed no suppression in the contralateral gland. Thus, asymmetrical PA with slightly left adrenal gland dominance could be considered. However, the recovery of blood pressure and hormone levels with negative CCT results after adrenalectomy supported left adrenal gland predominance. Although CR did not show the suppression of the nondominant adrenal gland as determined by the LI, the outcome of the adrenalectomy justified the validity of the LI. Although the negative result of CCT demonstrated the improvement of hormone abnormalities, the clinical outcome still seemed insufficient. The amlodipine dose increased after surgery, followed by the changes in PAC and PRA (Fig 2.). A mild APA-like lesion may have been in the contralateral adrenal gland. Therefore, regular follow-ups have been performed to check and control the hormone levels after the operation.

Recent findings have demonstrated that DNA methylation status at the CpG island in the promoter region of CYP11B2 regulates its expression (7). Studies have revealed that DNA methylation of CpG sites in the Ad1 and Ad5 elements in the promoter region of CYP11B2 inhibits the binding of the coactivator complex (CREB1, NGF1B, and NURR1) to its regions, inhibiting the coactivator complex from promoting the expression of CYP11B2 (2). Furthermore, hypermethylation promotes the binding of the corepressor complex (MECP2) to Ad1 and Ad5, which consequently suppresses the expression of CYP11B2 (2). A previous study also demonstrated the hypomethylation of CpG1(Ad1), CpG2(Ad5), and CpG3 in APA compared to the control: the mean value of methylation ratio of CpG1(Ad1), CpG2(Ad5), and CpG3 in APA were less than 20%, 50%, and 50%, respectively (8). In the present case, the DNA methylation rate at CpG sites near the Ad1 and Ad5 binding sites was higher than that in the previous report (CpG1[Ad1]: 75%, CpG [Ad2]: 100%), suggesting that CYP11B2 expression was suppressed (8). This result is consistent with negative CYP11B2 immunohistochemistry results but is inconsistent with APA characteristics. Furthermore, immunohistochemical analysis revealed a site of strong CYP11B2 expression in the vicinity of the adenoma, which was thought to be an APCC and might have been the responsible lesion.

The relationship between APA with the pathogenic variant in *KCNJ5* and the DNA methylation status of CYP11B2 has not been elucidated. Previous studies confirmed hypomethylation in the promoter region of CYP11B2 in APA compared to the normal adrenal tissue (2). However, a previous report demonstrated that APA-driving genes inducing aldosterone excess (ie, KCNJ5 and ATP1A1) did not relate to DNA methylation status in the CYP11B2 promoter region (9). Based on the present case, the pathogenic variant in *KCNJ5* did not cause hypomethylation in CYP11B2. Instead, CYP11B2 was methylated, which possibly led to the negative immunochemistry for CYP11B2. Other factors may have been involved in regulating DNA methylation in CYP11B2.

According to previous studies, nontumor unilateral PA had been reported in the literature (10). In the present case, APCC was detected through CYP11B2 immunostaining. However, the adenoma showed negative results for CYP11B2. This implied that APCC might have been the possible source of aldosterone excess. Nishimoto et al demonstrated the presence of nontumor lesions with small cell clusters named possible APCC to APA transitional lesions (pAATLs) by performing gene analysis and immunohistological evaluations (10). Although gene analysis and CYP11B1 immunostaining were not available because of limited resources, the APCC might have been a pAATL and could have been responsible for the lesion in the present case. However, APCC was detected in the healthy adrenal cortex by the donors (11). It was difficult to distinguish pAATLs and APCC without integrated immunohistochemistry.

Referring to the previous literature, the *KCNJ5* mutant nodule with negative CYP11B2 immunostaining is rare in PA. Xie et al reported positive immunostaining for CY11B2 in all the multiple nodules with pathogenic variants in *KCNJ5* (17/17), which implied a high prevalence of CY11B2 activation in nodules with pathogenic variants in *KCNJ5* (12). However, several studies reported *KCNJ5* mutant nodules with negative CYP11B2 immunostaining. A study reported by Sousa et al investigated the heterogeneity in CYP11B2 expression in adenomas with pathogenic variants in *KCNJ5* (13). The study revealed 2 kinds of adrenocortical adenomas in 1 adrenal tissue: adrenocortical adenoma with CYP11B2 activation or without activation (13). This could have been explained by the DNA methylation status. Likewise, in the present case, DNA methylation at the promoter region of CYP11B2 might have been observed in the CYP11B2-deactivated adenoma. Based on the results of immunohistochemistry in the present case and the previous study, some adrenocortical adenomas with pathogenic variants in *KCNJ5* may not be in charge of the lesion. Rather, aldosterone production could be suppressed. Thus, CYP11B2 immunohistochemistry should be conducted to accurately clarify the lesion of the unilateral PA.

In summary, we present a case of unilateral PA with adrenocortical adenoma. Although the pathogenic variant in *KCNJ5* was detected, CYP11B2 expression was suppressed, as confirmed by the negative immunohistochemistry results of CYP11B2 and methylation at the promoter region of CYP11B2. This intriguing case prompts further research into the effects of the relationship between DNA methylation and adrenocortical adenoma.

## Learning Points

- Adrenal tumors with pathogenic variants in *KCNJ5* do not always have a strong CYP11B2 expression.
- The aldosterone synthesis could possibly be suppressed by the DNA methylation of CYP11B2 in unilateral PA with adrenocortical adenoma.
- The criteria for AVS subtyping are still controversial. Postoperative evaluation, including pathological findings and clinical outcomes, should be confirmed to validate the AVS result.

## Data Availability

Restrictions apply to the availability of some or all data generated or analyzed during this study to preserve patient confidentiality or because they were used under license. The corresponding author will on request detail the restrictions and any conditions under which access to some data may be provided.
